# piRNAs Regulated by Mitochondria Variation Linked With Reproduction and Aging in *Caenorhabditis elegans*

**DOI:** 10.3389/fgene.2020.00190

**Published:** 2020-03-25

**Authors:** Zuobin Zhu, Ying Li, Mengyu Liang, Lei Wang, Liang Wang, Joshua D. Rizak, Conghui Han, Wenda Zhang

**Affiliations:** ^1^Department of Genetics, Xuzhou Medical University, Xuzhou, China; ^2^Medical Technology School of Xuzhou Medical University, Xuzhou, China; ^3^Department of Bioinformatics, School of Medical Informatics and Engineering, Clinical College of Xuzhou Medical University, Xuzhou, China; ^4^Department of Histology and Embryology, Xuzhou Medical University, Xuzhou, China; ^5^Department of Biochemistry, Xuzhou Medical University, Xuzhou, China; ^6^R.A.S. Innovation, Regina, SK, Canada; ^7^Department of Clinical Medicine, Xuzhou Medical University, Xuzhou, China; ^8^Department of Urology, Xuzhou Central Hospital, Xuzhou, China

**Keywords:** *C. elegans*, mitochondria, piRNA, reproduction, aging

## Abstract

In *Caenorhabditis elegans*, the binding of Piwi protein to a non-coding RNA form, called piRNA, has been found to be important to both reproductive and aging processes. As the biosynthesis of piRNA is modulated by mitochondrial function, it is likely that the interaction between mitochondrial function and piRNA expression plays an unknown, yet important, role in reproductive and aging processes because both processes are known to be affected by declines in mitochondrial quality and activity. While the relationship between reproduction and longevity is not characterized in full, the optimality theory of aging and the disposable soma theory suggest that a trade-off between energy and resources is needed for reproductive and aging maintenance. In this study, the influence of mitochondrial variations, via a respiratory chain complex IV (COX1) polymorphism, on piRNA expression was examined in relation to the reproductive and aging outcomes of *C. elegans*. The COX1 polymorphism in mitochondria was found to affect the number of piRNAs expressed, the development of germ cells, and the length of the lifespan of the nematodes. Interestingly, more than two-thirds of the piRNA expression changes associated with the mitochondrial variation were found to also be affected by age. A gene ontology analysis of the altered piRNA species found that the piRNAs affected by mitochondrial variation and age were linked to genes known to have roles in reproductive and developmental function. Moreover, a piRNA-lncRNA-mRNA regulatory network based on the differential expression patterns of piRNA related to the mitochondrial variation was constructed to further identify potential gene targets with functional interactions. Similarly, this network identified genes involved in reproduction, development, and aging processes. These findings provide new insight into understanding how mitochondrial variations may regulate piRNA expression and may influence the underlying molecular mechanisms that affect reproduction and aging.

## Introduction

Piwi protein is specifically expressed in animal germ cells, regulating a series of reproductive-related events, such as reproductive stem cell self-renewal and maintenance and germ cell development and differentiation. In short, Piwi protein is essential for gametogenesis.

Piwi protein has been found to bind specifically to a recently discovered type of non-coding RNA, named piRNA. This interaction has been found to play an important role in germline DNA integrity (Klattenhoff and Theurkauf, 2008), embryonic development (Du et al., 2016), immune defense [2] and cancer prediction (Zhang et al., 2016), etc. Moreover, piRNA also plays an important, albeit unknown, role in evolution (Parhad and Theurkauf, 2019). Most piRNA sequences are not highly conserved, but their location in the genome is highly conserved (Girard et al., 2006). As reproduction plays such an important role in the process of evolution because it is the basis for the continuation of populations, the roles of Piwi in gametogenesis and the conserved locations of piRNA coding regions suggest the Piwi-piRNA pathway has an evolutionarily conserved role in the regulation of reproduction in animals (Bagijn et al., 2012).

Two development theories on reproductive evolution, the optimality theory of aging and the disposable soma theory, suggest there exist trade-offs between energy and resources for the maintenance of soma reproduction (Partridge and Barton, 1993). These trade-offs are well-described phenomena in relation to life history traits (Kozlowski and Stearns, 1989), where aging is an important trait in the life history of nearly all species. As such, a commonly studied trade-off is the relationship between aging and reproduction (Flatt, 2011). In recent years, studies have found that piRNA is closely related to aging-associated diseases, such as Alzheimer’s disease (Sun et al., 2018), which indicates that piRNA may serve as a bridge and bond between reproduction and aging.

The primary energy source necessary for both reproduction and life history traits related to aging is provided by the mitochondria. Mitochondria have their own genome (mtDNA) and the normal function of mitochondrial genes is a necessary condition for longevity. In fact, mtDNA variation or decreased mitochondria numbers have been found to accelerate the aging process. Similarly, mitochondria are essential organelles for reproductive processes such as cell proliferation. For example, daughter cells of stem-like cells that inherit younger mitochondria exhibit higher stem-like activity (Katajisto et al., 2015). Mitochondria dysfunction, on the other hand, is known to lead to reduced sperm quantity (Amaral et al., 2013), fertilization abnormalities, embryonic death, and premature ovarian failure (Tsai and St, 2018). All of these reproductive dysfunctions are important manifestations of reproductive system aging that are linked to the mitochondria.

The reproduction and lifespan of *Caenorhabditis elegans* is a convenient model for genetic analysis because a standardized and uniform environment can support different populations of live worms. In our previous studies, it was found that the lifespan and the fecundity of *C. elegans* were both influenced by mitochondrial polymorphisms (Zhu et al., 2015, 2019). The apparent differences between two wild-type isolates of nematode, the Bristol strain (N2) and the Hawaii strain (CB4856), were found to be related to mitochondrial physiological functions and a natural mitochondrial gene variant of the COX1 subunit of mitochondrial complex IV (CIV) (Dingley et al., 2014). The COX1 variation in the CB4856 strain had significantly increased CIV activity and mitochondrial membrane potential (Dingley et al., 2014). Our previous study also found that a nematode strain carrying CB4856 mitochondria, but a N2 nuclear genome, has a reduced mitochondrial O_2_ consumption rate (OCR) in comparison to the wild-type N2 strain in both young and old worms. The results suggested that the COX1 variation in the mitochondria can alter energy metabolism in the nematode.

The relationships between mitochondria variation and piRNA function are generally unknown, making appropriate studies into this relationship of great value to understanding the interaction between reproduction and aging. In this study, a wild-type N2 strain and a trans-nuclear hybrid *C. elegans* that was bred to have the CB4856 mtDNA genome in nematodes with the N2 nuclear genomic background were used to examine how natural variations of COX1 in mitochondria interact with piRNA expression and function and thus influence the reproduction and the lifespan of *C. elegans.* In brief, this study provided an insight into the roles of COX1 variations on reproduction and the aging process through piRNA functions in *C. elegans* and identified important potential reproduction and aging targets for future studies directed at intervening in reproductive dysfunction and preventing age-related disease progression.

## Materials and Methods

### Strains and Media

N2 and CB4856 wild-type *C. elegans* strains were purchased from the Caenorhabditis Genetics Center (CGC) of the University of Minnesota. The NC30 strain was generated by crossing N2 males with CB4856 hermaphrodites and then repeatedly crossing the offspring with N2 males for up to 20 generations. All *C. elegans* were cultivated at 20°C on nematode growth medium seeded with *E. coli* OP50.

### Sperm Count Measurement

Sperm measurement was performed as previously described (Dai et al., 2019). Five synchronized L4 stage worms were placed onto a microscope slide with 10 μL M9 buffer. The worms were washed three times with 90% ethanol. In the dark, 10 μL of 0.2-μg/mL DAPI stain was then added to the slide and covered with a coverslip. After 5 min of incubation in the dark, slides were observed under a dark microscope. The fine particles with blue luminescence are sperm.

### Lifespan Brood Size Measurement

20 synchronized L4-stage worms were placed onto NGM plates seeded with *E. coli* OP50. Then, the worms were allowed to lay eggs in a new NGM plate on each day. The eggs in each dish were counted.

### Motility Assay

Synchronized worms were separately transferred to 3-ml plates with food-free NGM medium and habituated for 3 min at 20°C. Then, a CCD camera was used to continuously record the motility of the worms in M9 buffer. The number of head sways from left to right over 30 s was recorded. All strains of worms used in this study were examined at the same stages and each experiment was repeated three times.

### Total RNA Extraction and Small RNA-seq Data Analysis

Samples were collected from N2 and CN30 worms at young (day 0) and old adults (day 12) stage. RNA was extracted using Trizol (Invitrogen, Carlsbad, United States) according to the manufacturer’s instructions. Small RNA libraries were qualified via pair-end sequencing on the BGISEQ-500 platform (BGI-Shenzhen, China) (Hou et al., 2019). The following contaminant tags and low-quality tags from the FASTQ files of the small RNA sequences were removed to get clean tags in the final data. The impurities removed from the raw data included 5′ primer contaminants, insert tags, oversized insertions, low-quality tags, poly A tags, and small tags, among which the insert tags and 5′ primer contaminants were defined as adaptor contaminants, oversized insertion manifests, or as missing 3′ primers. The clean data that were able to be compared to the piRBase database, i.e., without any mismatches, were annotated as piRNA and/or piRNA expression as well as those not annotated to the miRBase and Rfam databases. The piRNA expression level is calculated by using Transcripts Per Million (TPM) (t Hoen et al., 2008).

### Gene Ontology (GO) Analysis

Gene ontology (GO) analysis was used to divide the functional modules of the genes. If the candidate gene was significantly enriched in a functional module, the proportion of the candidate genes was significantly higher than the overall genes in the functional module. According to the classification of the go_p annotation, a phyper function in the R software was used for enrichment analysis and a P value was calculated.

### piRNA Target Prediction and Gene Regulatory Network Construction

Multiple software packages were used to find possible piRNA targets. Intersecting targets were taken under appropriate filter conditions, such as minimum free energy (MFE), to generate a score for further analysis. Generally, RNAhybrid (Kruger and Rehmsmeier, 2006) and miRanda (John et al., 2004) were used to predict a piRNA target. A piRNA–lncRNA and piRNA–mRNA interaction network was constructed and visually displayed using the CytoScape software (Shannon et al., 2003).

## Results

### Variation in COX1 Affects Spermatogenesis and Health-Span

**FIGURE 1:**
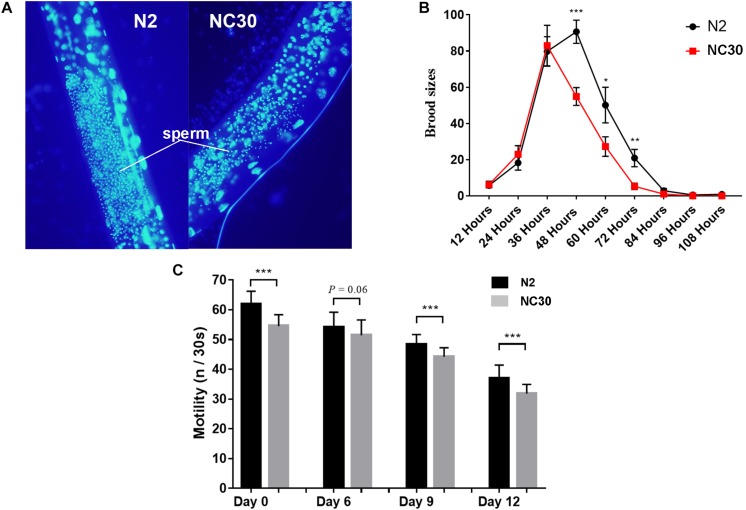
COX1 variation affects spermatogenesis and health span. **(A)** The amount of sperm in N2 and NC30 worms. The fine particles with blue luminescence are sperm. **(B)** The brood size of the N2 and NC30 worms. The brood size was detected every 12 h. **(C)** Motility ability changes with age. The motility of 0-, 6-, 9-, and 12-day-old worms cultured on the same NGM plate were examined (n represents head sways). Statistical analysis was performed using a two-tailed unequal variant Student’s *t*-test (***P-value < 0.001).

The trans-nuclear hybrid strain NC30, which carried CB4856 mitochondria (expressing a COX1 polymorphism) with a N2 nuclear background, was generated to determine whether the COX1 variation in CB4856 would reduce reproduction in nematodes with a N2 nuclear background. It was found that the sperm number of NC30 was significantly lower than the wild-type N2 worms (Figure 1A). In addition, the number of fertilized eggs of NC30 worms was also significantly lower than in the wild-type N2 strain. Moreover, the downward trend in the number of eggs that coincides with age in the NC30 worms was more pronounced than in N2 worms (Figure 1B).

Our previous study showed that the NC30 nematodes have a reduced life span in comparison to N2 worms (Song et al., 2020). Motility is an important physiological and functional indicator of health-span (Bansal et al., 2015). In this study, the motility of 0-, 6-, 9-, and 12-day-old *C. elegans* was quantified according to liquid media spontaneity in a wave-like channels assessment. The motility of NC30 worms was significantly lower than the motility of N2 worms during their whole lifespan (Figure 1C). This result suggested that the variation of COX1 can influence reproduction and health-span.

### Variation in COX1 Affects piRNA Profiles

**FIGURE 2:**
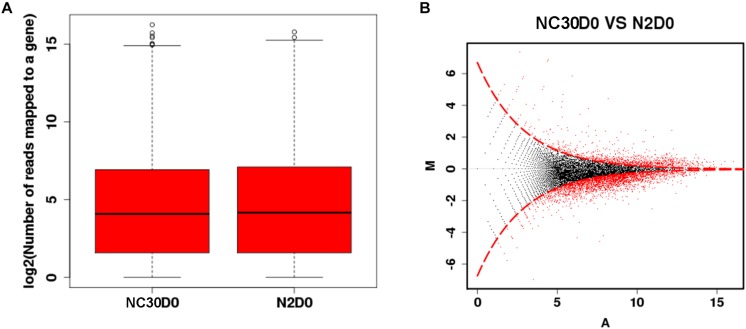
The piRNA profiles affected by COX1 variation. **(A)** Whole-genome piRNA expression between N2 and NC30. **(B)** MA plot for the differential expression of piRNA. The vertical axis (M) shows the intensity ratio between the N2 and NC30 for the same data point, while the horizontal axis (A) shows the expression level of piRNA. For the vertical axis, the points below horizontal line at 0 means the piRNA has a low expression in NC30 worms. The red dots represent the differentially expressed piRNAs.

The effect of mitochondria variation on piRNAs and their influence on the reproduction of *C. elegans* was evaluated by comparing the piRNA profile of NC30 and N2 worms with the same nuclear background, but different mitochondrial genotypes. The influence of the COX1 variation on whole-genome piNRA expression levels was first assessed between N2 and NC30. There were no significant differences in the average piRNA expression level between N2 and NC30 within the 15,920 piRNA sequences detected (Figure 2A). Then, different piRNA transcripts between NC30 and N2 worms were individually screened. piRNAs meeting a threshold value indicating a > 2 fold-change in transcript levels and a *q*-value ≤ 0.001 were identified as having significantly different expression levels. The expression of 975 piRNAs was significantly altered in the comparison of NC30 nematodes with N2 controls (Figure 2B). Among these piRNAs, 817 piRNAs were down-regulated and 158 piRNAs were up-regulated in the NC30 nematodes. The differentially expressed piRNAs are listed in Supplementary Table S1.

### Age-Dependent Changes in piRNA Expression

**FIGURE 3:**
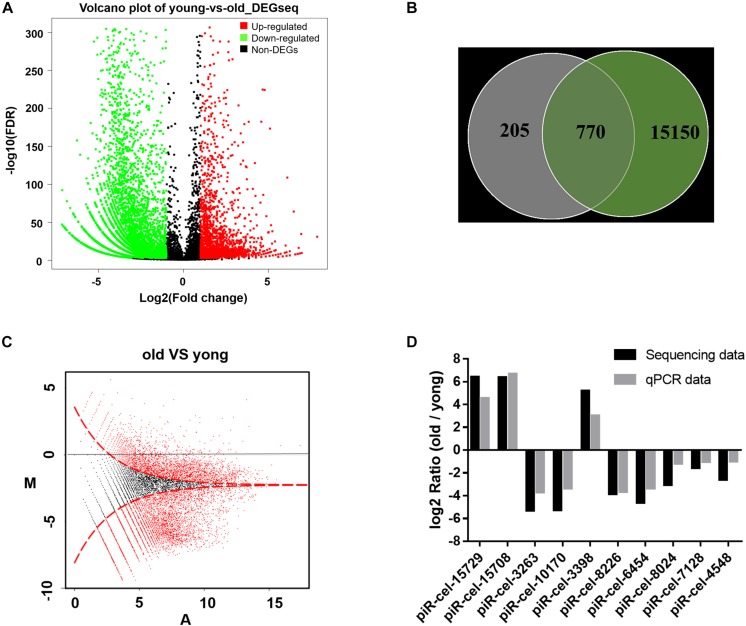
Age-dependent changes in gene expression levels. **(A)** Distribution of differentially expressed piRNA. The x axis is the log2(Fold change) of differentially expressed piRNAs, the Y axis is the –log10 (FDR) of differentially expressed piRNAs, with green points indicating down-regulation [log2(Fold change) ≤ –1 and FDR ≤ 0.001] and red points indicating upregulation [log2(Fold change) ≥ 1 and FDR ≤ 0.001]. **(B)** Venn diagram of the differentially expressed genes. Each circle represents a set of piRNAs. The left circle represents the piRNAs associated with mitochondria variation. The right circle represents the piRNAs associated with aging. The region superimposed by circles represents the intersection of piRNAs, both associated with mitochondria variation and aging. **(C)** MA plot for the differential expression of piRNA. The horizontal axis (A) shows the expression level of piRNAs, while the vertical axis (M) shows the intensity ratio between the young and old worms for the same data point. For the vertical axis, the points below the horizontal line at 0 mean the piRNA has a low expression in old worms. The red dots represent differentially expressed piRNAs. **(D)** piRNA sequencing data qPCR validation. piR-cel-15729, piR-cel-15708, piR-cel-3263, piR-cel-10170, and piR-cel-3398 were the largest expression differences piRNA; the other 5 piRNAs were the randomly selected piRNAs from the 8,183 piRNAs.

Genes affected by the mitochondrial variation were also examined to determine whether they also influenced aging. A previous study showed that piRNA could be altered during aging in *C. elegans* (Kato et al., 2011; Kim and Lee, 2019). In this study, in older worms, more genes showed a lower expression level of piRNAs than in developing worms. 8,183 piRNAs were identified to have significantly differential expressions between young and older worms (Figure 3A and (Supplementary Table S2). 79% of the piRNAs that were associated with the mitochondrial variation showed age-related expression features (770/975 piRNA associated to COX1 variation vs. 8,183/15,920 total piRNAs, *P-*value < 0.0001, two-tailed Fisher exact test) (Figure 3B). It was found that variation in COX1 not only reduced the reproduction and health-span of *C. elegans*, but also reduced the overall expression of piRNAs (Figure 3C). To validate the accuracy and reliability of the quantified piRNA sequencing data, the top five piRNAs (identified with the largest expression differences) and five randomly selected piRNAs from the 8,183 piRNAs for quantitative real-time polymerase chain reaction (qPCR) analysis. The quantified sequence data was consistent, with respect to the expression levels of the piRNA, with the qPCR results (Figure 3D). Together, these results show that piRNAs expression decreased significantly with age and suggest that the role the mitochondrial COX1 variation has in regulating reproduction and aging may also influence piRNAs levels related to these processes.

To determine what functions may be influenced by expression level changes, a GO term analysis identifying the functional annotation of potential target mRNAs was conducted, because piRNAs play a biological function by targeting corresponding mRNA sequences. The GO analysis indicated that the target mRNAs of the piRNAs affected by the COX1 mitochondria variation were predominantly related to developmental processes and reproduction function (Table 1). In addition, the target mRNAs of piRNAs affected by the COX1 mitochondrial variation also identified mRNAs known to influence aging. These mRNAs were related predominantly to metabolic processes, developmental processes, and reproduction function (Table 1). These results suggested that piRNA may play an important, albeit undefined, role in the regulation of reproduction and aging.

**TABLE 1:** The top 10 Gene Ontology terms identified for the differentially expressed piRNAs.

N2 vs. NC30		Young vs. Old	
	
Gene Ontology term	*P*-value	Gene Ontology term	*P*-value
Anatomical structure development	1.57e−37	Macromolecule metabolic process	9.29e−28
Developmental process	5.90e−37	Nitrogen compound metabolic process	1.08e−27
Multicellular organism development	1.66e−35	Primary metabolic process	2.54e−26
Reproductive process	7.98e−35	Locomotion	7.35e−26
System development	1.00e−34	Negative regulation of biological processes	1.84e−25
Negative regulation of biological processes	1.59e−32	Organic substance metabolic process	4.06e−24
Reproduction	4.82e−32	Reproduction	4.17e−22
Locomotion	3.26e−30	Cellular component organization	4.71e−22
Cell junction organization	5.43e−30	Developmental process	6.29e−22
Anatomical structure morphogenesis	6.12e−30	Reproductive process	2.37e−21

### Interactive Network of piRNA-lncRNA-mRNA

**FIGURE 4:**
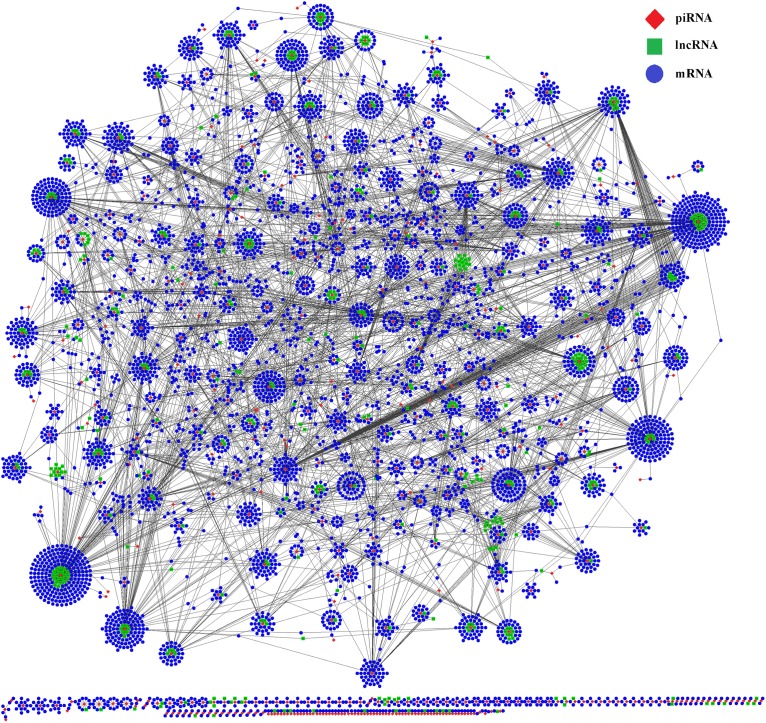
Interactive network of piRNA, lncRNA, and mRNA. The regulatory network was constructed through visualizing the relationships between differentially expressed piRNAs and their target genes. The regulatory network consists of 496 piRNAs, 5,834 mRNAs, and 459 lncRNAs.

To better understand the functions of piRNAs and the corresponding genetic regulatory networks affected by COX1, a piRNA-lncRNA-mRNA network was visualized with the software CytoScape (Shannon et al., 2003). The mRNA targets for the piRNAs affected by COX1 were predicted (Supplementary Table S3) and the relationships between piRNA and lncRNA were also determined by the position of the piRNA and lncRNA on their respective chromosomes (Supplementary Table S3). Then, complex regulatory networks were generated according to the interactions between differently expressed piRNAs and their targets RNAs (Figure 4). These modeling results indicated that the mitochondrial variation influenced reproduction and aging processes through a complex network. In this network, it was identified that lncRNA and mRNA are potential targets of piRNAs, that most piRNAs target more than one mRNA or lncRNA, and that the effects of the network are not mitigated through a single piRNA.

## Discussion

The Piwi–piRNA pathway has been evolutionarily conserved in many germlines (Bagijn et al., 2012). In *C. elegans*, piRNAs are about 20 nucleotides in length and have a 5’ uracil nucleotide end (Ruby et al., 2006). Previous studies have suggested that piRNAs provide heritable, sequence-specific triggers for RNA interference in *C. elegans* (Bagijn et al., 2012). Interestingly, most of these previous studies on piRNAs focused on reproduction, and a few studies were related to aging (Kim and Lee, 2019). Moreover, studies have confirmed that mitochondria function is required for Piwi-interacting RNA (piRNA)-mediated silencing of transposable elements in fly and mouse germlines, suggesting that mitochondria play an important role in piRNA pathway (Aravin and Chan, 2011). Thus, piRNAs may play a link role between mitochondria and reproduction and aging. In this study, *C. elegans* was used as a model organism to explore the relationship among mitochondria, piRNA, reproduction, and aging.

Since the piRNA pathway has been found to be influenced by mitochondria and is involved in the regulation of reproductive function and aging, it was hypothesized that mitochondria influence reproduction and aging through piRNAs. Mitochondrial genes account for only a very small part of an organism’s genome, but the mitochondria provide the necessary energy for cellular function and play an important role in signal transduction, cell growth, and apoptosis. Our previous studies had shown that the degree of mismatch between the mitochondria and nuclear genome may be an important factor that influences reproduction and lifespan differences among *C. elegans* populations (Zhu et al., 2015, 2019). These earlier studies included the construction of a nuclear transfer *C. elegans* germline containing a COX1 mitochondrial gene variation and the same genomic background as wild-type nematodes (Bristol Strain) and found the germline to have reduced mitochondrial function relative to N2 *C. elegans* and to have reduced function related to reproduction and reduced lifespans. In this study, a similar nuclear transfer line of *C. elegans* carrying the N2 nuclear genome and CB4856 mitochondria was used to compare the effects of mitochondrial variation on piRNA expression. The investigation in this study focused on changes in piRNA gene expression influenced by the natural COX1 variation of mitochondria within *C. elegans* and found that a number of piRNAs were affected by the COX1 variation and aging.

Aging is generally accompanied by a decline in biological function, especially with respect to longevity and motility. Here, changes with respect to health-span/motility were also studied in the NC30 *C. elegans* construct and found that the natural COX1 variation in the mitochondria both influenced the health-span in the nematodes and the piRNA gene expression levels. The expression level changes for a number of piRNAs were found not only to be affected by the COX1 variation, but also to have expression changes that coincide with aging. As our previous study demonstrated that this strain of nematode also had reduced lifespans in comparison to wild-type nematode (Song et al., 2020), the indirect correlations between COX1 mitochondrial variation, reproduction, aging, and piRNA expression/function suggest that the interaction between the mitochondrial variation and piRNA expression need further study to fully understand their direct influence over reproductive and/or aging processes.

To better understand the biological functions and potential mechanisms by which COX1 affects piRNA expression and how this influence may affect reproduction and aging, a GO analysis was performed and used to identify potential down-stream targets of piRNA function. Among the GO terms found in this study, potential reproduction and development processes linked to piRNA sequences were identified to be both influenced by mitochondria variation and age in the nematodes. In addition, piRNA–lncRNA and piRNA–mRNA interactions were also identified from the same sample of piRNA, mRNA, and lncRNA expression data of the nematodes. The construction of a piRNA–lncRNA–mRNA interaction network predicted similar piRNA functions and provided a genetic model for the effects of the mitochondrial COX1 variation to potentially influence reproduction and aging through piRNA signaling. Collectively, this experimental and computational modeling study provided new evidence directed at understanding how mitochondria may influence molecular mechanisms that govern reproduction and aging, as well as suggested that the piRNAs may have an important, albeit undefined, role in those reproductive and aging mechanisms. As such, it is expected that future research or piRNAs will uncover new mechanisms involved in reproductive and aging processes, if not serve as new biomarkers in future reproductive and aging research.

## Data Availability Statement

All data needed to evaluate the conclusions in the paper are present in the paper and/or the Supplementary Materials. The transcriptome raw data are available at GEO (Accession Number: GSE138352).

## Author Contributions

ZZ, CH, and WZ designed and wrote the manuscript. ZZ and YL conceived the experiment, analyzed, and interpreted the data. ML and JR conceived of the experiment. All authors discussed and commented on the manuscript and agreed to be accountable for all aspects of the work in ensuring that questions related to the accuracy or integrity of any part of the work are appropriately investigated and resolved.

## Conflict of Interest

JR was employed by R.A.S. Innovation. The remaining authors declare that the research was conducted in the absence of any commercial or financial relationships that could be construed as a potential conflict of interest.
